# The Interplay of Religion and Singlehood: A Qualitative Study of Christian Single Adults Living in Poland

**DOI:** 10.1007/s10943-025-02345-z

**Published:** 2025-06-06

**Authors:** Sebastian Pietrzak, Katarzyna Adamczyk, Beata Zarzycka

**Affiliations:** 1 Faculty of Psychology and Cognitive Science, Adam Mickiewicz University, Poznań, Poland; 2 Institute of Psychology, The John Paul II Catholic University of Lublin, Lublin, Poland

**Keywords:** Psychology of religion, Religious attributions, Religious coping, Singlehood, Well-being

## Abstract

Previous research has suggested that religion and spirituality may play positive and negative functions in single people’s lives. However, while prior studies have provided initial findings, a more comprehensive and thorough exploration of the role of religion in the experience of singlehood is required. To address this gap in the literature, qualitative interview-based research was performed with a sample of 30 single Christian adults living in Poland aged 19–63 years (*M* = 35.37; *SD* = 10.27), namely, 16 women and 14 men. The reflexive thematic analysis identified three major themes: 1) religion as a source of negative experiences associated with singlehood, 2) religion as an aid in mitigating negative experiences associated with singlehood, and 3) religion as a criterion for choosing a life partner and the foundation of an intimate relationship. The identified themes imply that the functions of religion in the experience of adult singlehood may reflect religious attributions employed by single individuals to make meaning of their single status, regain a sense of control in their lives and preserve positive self-esteem while remaining single. The findings highlight the importance of religion in interpreting the determinants, nature, and outcomes of singlehood among single Christian individuals.

## Introduction

The share of single adults that is, adults who currently do not have a lifetime partner/spouse and are not in a marital or informal relationship (DePaulo & Morris, 2005), has increased in recent decades in many countries, including the USA, Canada, and Asian and European countries (Girme et al., 2023). This trend has led to heightened efforts among researchers to investigate the factors affecting the functioning of single individuals more thoroughly (e.g., Girme et al., 2023; Ochnik & Adamczyk, 2025). Notably, Girme and colleagues (2023) have identified religion and religious communities as unexplored areas within the singlehood literature. However, scholars have recently begun to intensively investigate the role of religion in single adults’ lives (Adamczyk et al., 2024a, 2024b, 2024c; Himawan et al., 2024; Lianda & Himawan, 2022), extending previous studies in this area (e.g., Granqvist & Hagekull, 2000; Himawan et al., 2018a, 2018b; Himawan, 2020a, 2020b).

Prior studies have demonstrated, for example, that single university students report greater religious activity, a greater personal connection with God, a stronger focus on religious beliefs, and increased religiosity related to emotional regulation than their coupled peers (Granqvist & Hagekull, 2000). Studies by Himawan (2020a) and colleagues (2018a; b) have shown that attachment to religious values and rituals may serve as a coping method with singlehood. Additionally, higher levels of religiosity can be associated with higher levels of life satisfaction among unmarried individuals (Himawan, 2020b; Lianda & Himawan, 2022). The positive functions of religion in single people’s lives also involve providing a meaning of singleness, whether by perceiving singlehood as a temporary period willed by God, as an opportunity to embrace life beyond marriage, or as a period during which conservative religious norms are negotiated with contemporary values (Himawan, 2020b). In addition, religion may not only represent a source of a sense of belonging and a close spiritual relationship with God but also constitute the religious community (Himawan, 2020b).

Finally, recent studies performed in predominantly Roman Catholic Poland have revealed additional functions of religion in single people’s lives. Adamczyk and colleagues (2024c) described six themes drawn from qualitative interviews with single adults, demonstrating how religion directly relates to their experience of singlehood: (a) religion as a criterion in selecting a partner/spouse and a guide in the relationship domain; (b) the church community as a potential community for finding a partner/spouse; (c) giving up control of one’s singlehood to God to find a partner/spouse; (d) redefining singlehood through religion as a benevolent and advantageous situation; (e) the internal struggle between religious values and desires in the domain of romantic relationships; and (f) confusion and dissatisfaction in one’s relationship with God.

Further, Adamczyk and colleagues (2024a) examined the temporal associations between spiritual well-being and satisfaction with singlehood. They revealed that greater spiritual well-being may be an important, although weak, factor related to higher levels of satisfaction with singlehood. Adamczyk and colleagues (2024b) also investigated the role of prayer for single adults. They demonstrated that confession prayer is related to decreased psychological well-being among single individuals who pray (Adamczyk et al., 2024b).

The abovementioned research has significantly contributed to the recognition of the relevance of religion and spirituality in single people’s lives and associated life outcomes. However, it remains unclear what specific functions of religion could be related to psychosocial functioning among single adherents. Therefore, to further examine in depth the role of religion in the experience of singlehood among single religious adults, in the current investigation, we conducted exploratory qualitative interview-based research with a sample of 30 Christian single adults living in Poland. This study design enabled us to generate new knowledge on this still little explored topic and formulate the following broad, open research question: “How do Christian single adults relate to religion, and what functions does religion fulfill in their experience of singlehood?”.

Given the exploratory nature of this investigation, we utilized a conceptual framework instead of a theoretical one, following the perspective of Miles and colleagues (2020). They argue that this method offers “a more inductively derived and evolutionary model” (p. 15) that encompasses “case-specific variables, concepts, context, and participants” (p. 15) while also being grounded in the unique elements of a specific study. The conceptual framework of our investigation is built on two main components. First, it considers the general functions of religion in people’s lives, which have been recognized in extant literature and prior single studies that have examined the links between singlehood and religion. Second, it acknowledges the potential impact of Christianity and the Polish marital and religious contexts in which single individuals function.

### The Role of Religion in People’s Lives

Religion is a complex construct that includes structural and functional dimensions (Hill & Edwards, 2013; Tsang & McCullough, 2003). The structural aspect involves the contents of religiosity—beliefs, practices, and personal traits—that help shape what defines an individual as being religious. The functional aspect defines how religion influences an individual’s life by shaping their motivations, coping strategies, and emotional well-being (Hill & Edwards, 2013). This aspect includes emotional regulation, prayer, religious coping, and spiritual struggles. For instance, the structural approach based on relational theories of religiosity, including attachment theory and religious attribution theory (Hill & Edwards, 2013), may help analyze how single individuals engage in religion. Attachment theory highlights the emotional bond and interactive dynamics with God (Kirkpatrick, 2005); this approach can help analyze whether God serves as a secure base or a compensatory attachment figure in the absence of a romantic partner. Religious attribution theory focuses on the cognitive interpretation of divine influence in one’s life (Spilka et al., 1997). It can help examine how individuals perceive their singlehood within a religious framework.

The functional approach can serve in analyses of the role of religion for single individuals by examining its function in terms of coping (Pargament, 2010) and generating meaning (George & Park, 2016). By examining how religion helps with stress management and finding meaning through faith, this approach may demonstrate how religion supports single individuals in their singlehood through overcoming challenges, handling emotions, uncovering purpose, and integrating their experiences into a unified life story.

We recognized that both structural and functional approaches may provide valuable insights into the experiences of single people: The structural approach may focus on individuals’ beliefs, while the functional approach may examine how these beliefs influence their experiences of being single.

### Potential Links Between Christianity and Singlehood

In Christianity, marriage is considered a significant institution, and Catholic doctrine views it as established by God himself (Catholic Church, 1997, §1603). Catholic teachings suggest that singlehood can also be divinely inspired when lived in a certain way (Catholic Church, 1997, §1618–1620). From this perspective, singles (excluding priests and nuns) need to carry out the daily responsibilities of all Christians, aiming for holiness (John Paul II, 1988). Moreover, Christianity teaches that there is life after death, during which individuals do not marry (New International Version, 2011, Matthew 22:30). Thus, within the Christian faith, the separation between single and married individuals is viewed as temporary. Nevertheless, single believers may sometimes struggle with uncertainty—wondering whether their current life aligns with God’s will and whether their spiritual state is “good enough” without a traditional family path.

Furthermore, Christianity establishes norms in the domain of intimate relationships. Divorce is not recognized in Catholicism, and the only way to live with a partner in one household without committing sin is through a church-sanctioned marriage (Catholic Church, 1997, §1660–1662). In contrast, Orthodox Christianity and most Protestant denominations also discourage divorce but allow it under certain circumstances, which may provide greater flexibility for singles who are considering marriage with a divorcee. Additionally, all Christian traditions emphasize sexual restraint before marriage, although they differ in the extent to which they stress the necessity of adhering to this principle (Piwko, 2013).

Roman Catholicism, Protestantism, and Eastern Orthodoxy are all forms of community-based Christianity (Mathras et al., 2015). Consequently, single Christians, even without a lifelong partner or spouse, often experience a sense of belonging to a larger community. This connection can help fulfill their need for affiliation and social support, as religion can enhance well-being by fostering social integration and greater participation in both formal and informal social networks (e.g., Okruszek et al., 2022).

Finally, another important element of Christian doctrine, which may be important for single individuals, involves the belief in a personal God who actively participates in not only the world but also the lives of people (Ciesielski, 2015). As a result of this belief, it is plausible that single individuals may tend to interpret singlehood in reference to God’s actions, for instance, as a part of God’s plan or a period God places them in to prepare them for entering into a romantic relationship.

### The Polish Religious and Marital Contexts

In Poland, religion—particularly Catholicism—plays a central role in shaping the cultural landscape (e.g., Żemojtel-Piotrowska et al., 2023). A vast majority of Poles (78%) identify as religious, and among them, nearly 90% report affiliation with the Catholic Church, making Poland one of the most religiously homogeneous societies in Europe (Centre for Public Opinion Research, 2024; Zarzycka et al., 2017). Traditional social values also remain prominent: Polish society demonstrates a strong attachment to heterosexual marriage, widespread disapproval of divorce, and low acceptance of alternative family forms (Janicka & Szymczak, 2019). Public opinion polls indicate that 45% of Polish residents hold unfavorable views toward singlehood (Centre for Public Opinion Research, 2019). This religious alignment persists among singles: 88.7% describe themselves as religious, and 98.3% identify as Roman Catholic—the highest proportion in Europe (Adamczyk & Trepanowski, 2023).

At the same time, despite this high level of declared religiosity and the prevalence of traditional norms, research suggests that Catholic teachings do not always exert a strong influence on personal decisions and everyday behaviors—especially among younger generations and in more secularized urban settings (Mąkosa & Adamczyk, 2024). Areas such as sexuality, interpersonal relationships, and marriage choices often reflect a growing divergence between institutional religion and lived experience (Zarzycka, 2009). This complexity of contemporary Polish religiosity created an excellent opportunity in the current investigation to examine whether and to what degree individuals relate to religion in the situation of remaining single.

## Method

### Transparency and Openness

The dataset generated and analyzed in the present study is not publicly available to maintain the individual privacy of the participants; however, it is partially available from the corresponding author upon reasonable request. The entire interview schedule used in the investigation is available at the Open Science Framework [https://tinyurl.com/4y7ywxer]. The study was not preregistered.

### Researcher Reflexivity

In line with the guidelines for reporting qualitative research (Braun & Clarke, 2025), which highlight the researcher subjectivity as a resource for research, we acknowledge our training, research, and research values. These guidelines also emphasize the interpretative nature of reflexive thematic analysis (Braun & Clarke, 2023). We consider how our training and values might have shaped the performance of the analysis and interpretation of the results.

All three authors are psychologists, among whom the first and second authors specialize in single studies and adhere to the latest research line emphasizing the need to recognize factors mitigating the negative outcomes of adult singlehood. These two authors also assume in the literature that singlehood for some individuals may relate to positive or negative outcomes and be a source of distress or thriving (see, e.g., Grime et al., 2023). Consequently, the authors may have been inclined to interpret the findings to discover the manifestations of utilizing religion as a buffering means in the experience of singlehood, particularly when it is associated with adverse outcomes.

The third author is an expert in the psychology of religion, which may have influenced interpretation through increased sensitivity to the existential and symbolic aspects of the narratives of the participants. From a reflexive perspective, this perspective may have highlighted the role of religion not only as a coping mechanism, but also as a source of meaning. The interdisciplinary composition of the team allowed for a more balanced and critically reflective analysis.

The three authors received training in both qualitative and quantitative methods. Two authors who specialize in single studies developed their skills in performing qualitative interview-based research and reflexive thematic analysis at the stage of preparing their doctoral theses and the postdoctoral stage. This background and training have taught the authors to define qualitative research predominantly through the theoretical lens of singlehood. In turn, the third author, who specializes in the psychology of religion, approaches qualitative inquiry with a focus on meaning-making processes and the role of spirituality in human development. This perspective informed the analytical process by drawing attention to the deeper symbolic structures present in the accounts of the participants. As a result, the collaboration between authors with distinct yet complementary expertise contributed to a richer interpretation of the data, combining the relational, existential, and spiritual dimensions of singlehood.

Finally, all three authors were raised and are familiar with the religious and marital Polish contexts that are predominantly Roman Catholic and still strongly orientated toward traditional marriage and family. As a result of this shared background, the authors may make the assumption that religion and marital relationships are interconnected and represent important domains in adulthood, domains to which individuals actively relate and seek meaning within.

### Research Design

To investigate the experiences and personal narratives of single adults in Poland, we conducted qualitative research. This approach enables researchers to reveal subjective meanings and personal interpretations (Creswell, 2012). We used semi-structured interviews for their flexibility in capturing diverse perspectives, all while following a structured analysis framework (McGrath et al., 2018).

Our approach is grounded in a social constructivist epistemology, emphasizing the collective construction of meaning through personal experiences and social environments. Individuals create meaning both independently and socially, with knowledge being constructed rather than uncovered (Savin-Baden & Howell-Major, 2013). This process of meaning-making reflects a desire to understand the world (Creswell, 2012), with the researcher’s role being focused on exploring constructed meanings and understanding how they develop (Savin-Baden & Howell-Major, 2013). Simultaneously, the researcher recognizes the complexity of perspectives without trying to classify or normalize them, instead formulating broad, general questions while concentrating on the context of the study participants’ lives (Creswell, 2012). The constructivist approach is especially relevant for investigating lesser-known phenomena and their associated variables (Bäcker et al., 2016).

### Participants and Sampling

The current investigation was performed in a sample of 30 participants. This number was determined on the basis of literature recommendations pertaining to (1) saturation, which requires a larger number of interviews (i.e., from 16 to 24 interviews) (Hennink et al., 2017), and (2) information power, which considers a) the purpose of the study; b) the specifics of the sample; c) existing theories; d) the quality of the interviews; and e) the method of analysis used (Malterud et al., 2016).

This study employed maximal variation sampling, which is a purposeful sampling strategy in which participants vary with respect to some characteristics or traits (e.g., different age or sex groups) (Creswell, 2012). This strategy allows the exploration of multiple perspectives of individuals to demonstrate the complexity of their personal experiences (Creswell, 2012). The participants were recruited via posting flyers in Facebook groups for believers of Christian denominations. All participants were compensated for their participation in the interviews and received a shopping voucher worth 150 PLN (approximately 37 USD) to one of a Polish discount store.

The current investigation was targeted at non-clergy persons, and the main inclusion criteria were as follows: (1) being single, that is, not currently having a lifetime partner/spouse and not being in a marital or informal relationship; (2) religious affiliation, that is being a follower of a monotheistic religion. Notably, the levels of participants’ religiosity or commitment were not assessed before the performance of the interviews. However, the content of the interviews allows us to claim that participants met the criteria of a religious person proposed by Sarouglu (2011); that is, they were characterized by belief in transcendent entities, emotional commitment to a relationship with the sacred, commitment to religious practices and moral conduct, and membership in a religious community; and (3) living in Polish territory and being fluent in the Polish language. Individuals who met the above-indicated three major inclusion criteria were eligible to participate in the interviews, which ensured the achievement of maximal variation sampling (Creswell, 2012) and enrollment of individuals characterized by different levels of desire for a romantic partner, relational history, or having or not a child/children.

The participants included 30 involuntary and voluntary single individuals (16 women and 14 men) aged 19–63 years (*M* = 35.37; *SD* = 10.27). Nineteen participants had been in a marital or informal relationship in the past, while 11 participants had never been in a relationship; three participants were divorced, and one was separated. Twenty-eight participants were Poles, while two participants were Ukrainians fluent in Polish. All the participants lived in Poland and were Christians. All the participants were cis-gender and heterosexual. The participants and the authors did not have any formal or informal relationships. The detailed participants’ sociodemographic and religious characteristics are presented in Table 1.

**Table 1:** Participant demographic characteristics (N = 30)

Name	Sex	Age	Singlehood length	Voluntary singlehood	Being married in the past	Having children	Religion
Penelopa	W	19	Always single	Yes	No	No	Protestantism
Paweł	M	24	Always single	Yes	No	No	Protestantism
Krystian	M	25	Always single	No	No	No	Roman Catholicism
Pamela	W	25	Always single	Yes	No	No	Protestantism
Kira	W	27	Always single	No	No	No	Roman Catholicism
Karina	W	28	Always single	No	No	No	Roman Catholicism
Kamil	M	28	10 years	No	No	No	Roman Catholicism
Kornel	M	28	Always single	Yes	No	No	Roman Catholicism
Kamila	W	29	4 years	Yes	No	No	Roman Catholicism
Kinga	W	29	4 years and 6 months	No	No	No	Roman Catholicism
Kryspin	M	29	Always single	No	No	No	Roman Catholicism
Kasjan	M	30	6 years and 6 months	Yes	No	No	Roman Catholicism
Klaudiusz	M	30	10 years	No	No	No	Roman Catholicism
Ksenia	W	31	1 year	No	No	No	Roman Catholicism
Konrad	M	32	1 month	No	No	No	Roman Catholicism
Karolina	W	34	8 months	No	No	No	Roman Catholicism
Grażyna	W	35	1 year	Yes	No	No	Greek Catholicism
Kazimiera	W	35	4 years and 6 months	No	No	No	Roman Catholicism
Kornelia	W	36	Always single	No	No	No	Roman Catholicism
Katarzyna	W	36	4 years and 6 months	No	No	Yes	Roman Catholicism
Krystyna	W	37	3 years	No	No	No	Roman Catholicism
Olaf	M	39	Always single	Yes	No	No	Orthodox
Karol	M	41	Always single	Yes	No	No	Roman Catholicism
Piotr	M	46	1 year and 6 months (marital separation)	No	Yes	Yes	Protestantism
Kajetan	M	47	1 years and 6 months	No	No	No	Roman Catholicism
Klara	W	47	3 years (divorce)	Yes	Yes	No	Roman Catholicism
Kazimierz	M	47	10 years	Yes	No	No	Roman Catholicism
Klaudia	W	50	15 years and 6 months	No	No	No	Roman Catholicism
Paulina	W	58	2 years (divorce)	Yes	Yes	Yes	Protestantism
Przemek	M	63	20 years (divorce)	Yes	Yes	Yes	Protestantism

W, woman; M, man

### Procedure

The data collection process was preceded by a positive evaluation of the study by the Research Ethics Committee of the University (blinded name for review) (decision number blinded for review). Informed consent was obtained from the participants.

The individual semistructured interviews were performed by the first author between February and August 2023. The interview schedule consisted of 10 open-ended questions to elicit narratives on topics concerning the experience of singlehood and religion (see the complete interview schedule in the online supplementary materials, which are available at https://tinyurl.com/4y7ywxer). The initial stimulus prompted the narrative: “*Please tell me about your life as a single person [what is it like for you to be single?], including how it used to be and how it is now, and how you see your future*.”

The study was conducted remotely via the Skype and Microsoft Teams applications. The duration of the interviews ranged from 12 to 99 min. The recordings of the interviews were subjected to denaturalized transcription, the purpose of which was to faithfully represent the content of the interviews, omitting such audio elements as filler words, pauses, or stammers, for example (Oliver et al., 2005). The transcribed interviews did not contain any information that might reveal the participants’ identities. The participants were assigned fictitious names that were distinct from their actual names.

### Reflexive Thematic Analysis

We used reflexive thematic analysis to examine the data, adopting an inductive approach to identify and interpret patterns in qualitative data (Braun & Clarke, 2020a, 2020b, 2023, 2025). The analysis adhered to the six-step framework initially proposed by Braun and Clarke (2006) and further developed by Clarke and Braun (2016). These phases consist of 1) becoming familiar with the data, 2) coding, 3) searching for themes, 4) reviewing themes, 5) defining and naming themes, and 6) writing. This methodology provides a flexible and iterative approach to analysis, allowing themes to naturally emerge from the data while ensuring rigorous and in-depth interpretation.

While the first author conducted the analysis to enhance quality control and a reflective attitude toward the data and analysis, the results obtained were audited by the second and third authors. The audit involved reviewing the themes, providing feedback on their comprehensibility, communicability, and clarity, helping to organize the themes into a meaningful whole, and, as a result, obtaining the final consensus on the identified themes.

## Results

The reflexive thematic analysis allowed us to construct three major meaning-based interpretative themes reflecting the role of religion in the experience of single Christian individuals living in Poland. A brief overview of the three themes is presented in Table 2.

**Table 2:** Summary and description of themes identified in the qualitative interview-based study

Theme	Description	Subthemes	Example quote
1. Religion as a source of negative experiences associated with singlehood	Religion is described as limiting personal choices and causing frustration in a life lived as a single. Difficulties are associated with the obligation to follow religious rules related to the construction of romantic and sexual relationships and the perception of a relationship with God as a relationship with someone incomprehensible, uninterested in a person’s well-being, but directing specific requirements for entering a relationship	Difficulties in adherence to religious rules	I am seeking new relationships [as a person in marital separation]. However, this religion confirms my various concerns about whether it’s okay. (Piotr)
		Difficulties in the relationship with God	I have this sense that the Lord God created people to be in relationships and to just if someone does not go the way of the priesthood, well, to go the way of marriage, parenthood, so I have this sense that I am a little bit off from that God’s design. (Kinga)
2. Religion as an aid in mitigating negative experiences associated with singlehood	Religion is described as a resource to help cope with the stress of singlehood and a sphere to find meaning in a single life. In turn, contact with God and the religious community can compensate for the lack of a partner	A supportive perspective on seeing God and the community	[I see God] as a friend, one with whom I can, I use the metaphor, that I drink coffee with Him to myself from time to time. (Kornelia)
		Questioning the duty of being in a romantic relationship	People who join monasteries or Catholic priests who live celibate lives also live as single, and as a result, I don’t think it matters to God that others are also singles. (Paulina)
		The meaning provided by religion in singlehood	3) I have received talents from God, and it follows from faith that I should make the best use of them, act in harmony with myself, and do what I am best at [instead of strenuously seeking a relationship]. (Krystian)
3. Religion as a criterion for choosing a life partner and the foundation of an intimate relationship	Religion is described as a lifestyle that shapes singles’ approach to romantic relationships. The need to find a partner with shared Christian values and the belief in God’s influence on the success of this search also stems from religion		Among those people I met who were potential partners for life, it was only actually two people who were able to get married Catholic at all and would want to, and the others were people who were either non-believers, were divorced, or were just in some problematic situation, that is, they were typically people I would not want to get involved with. (Karolina)

### Theme 1. Religion as a Source of Negative Experiences Associated with Singlehood

The first theme involves two subthemes that reflect single adults’ perception of religion as a factor contributing to negative experiences of singlehood. Single individuals recognize religion as something external to singlehood, which constitutes a foundation for negative interpretations of their single status and diminishes their self-esteem. According to the participants, religion limits their choices and causes frustration, primarily through the need to follow religious rules and perceived difficulties in their relationship with God.

#### Subtheme 1. Difficulties in Adherence to Religious Rules

This subtheme captures participants’ belief that adhering to religious rules related to romantic and sexual relationships makes singlehood a period of struggle. For example, 29-year-old, never-married Kamila has given up her search for a partner because she does not plan to have children, and the exclusion of having offspring prevents her from entering into a sacramental marriage in the Catholic Church. She prefers to remain single to avoid living in a relationship that does not meet Catholic principles. This is illustrated in her following statement:

I do not want parenthood; I do not want motherhood. This awareness is clear to me, and I came to this conviction. I did not see much point in looking for a relationship because a relationship, in the Catholic understanding of the term, is, by definition, supposed to be directed one day from the perspective of striving for marriage. [...] I have a desire to have someone very close to me, a desire for a close relationship with a man on an exclusive basis. But my current situation is decidedly incompatible with this desire.

A few different difficulties in religious and moral observance are experienced by Kajetan, a 47-year-old single man who has never married. He often doubts the point of sexual abstinence and is frustrated by the need to maintain it. In addition, he is frustrated by his sacrifices’ lack of visible results. At the same time, he is being held back by his professed principles (*I don’t know, throw it all to hell and indulge in some hedonism there? But, well, it’s the principles that I follow that inhibit me from doing so*).

#### Subtheme 2. Difficulties in the Relationship with God

The second subtheme includes descriptions of the singles’ relationship with God, which is attributed as one that makes life as a single difficult. According to the participants, God is perceived in a way that arouses anger toward Him for condemning people to remain single. In addition, the specific perception of God implies a sense of failure to meet God’s requirements regarding establishing a romantic relationship.

The words of 36-year-old Katarzyna, who is a never-married single mother, illustrate the difficulties mentioned above:

I have resentment, much resentment toward the Lord God for sure, although according to my faith, I remember, somewhere in the back of my mind, He watches over my life and that everything has its time, yes? But I have a lot of regrets, resentment, and anger that He has not arranged my life.

Katarzyna tries to believe that God has a plan for her life, although she does not understand it now. Seeing God as someone incomprehensible or unkind in the context of an individual’s consent to enter a relationship is sometimes linked to the general image of God as a person to be feared. Konrad (32, never married) discusses this perception of God. He mentions that the image of God as someone punishing and harsh took root in his perception during his youth. Currently, that image competes with the image of God as someone gentle. However, when God appears to Konrad as someone punishing, it is then easier for him to explain his single life or health problems as punishment for his sins (*I may have sinned in the past; maybe the fact that I have sinned in the past caused the fact that I am now a single person*).

### Theme 2. Religion as an Aid in Mitigating Negative Experiences Associated with Singlehood

The second theme involves three subthemes that reflect participants’ statements where they attribute a supporting role to religion for their singlehood. This theme reveals the aspect of religion as coping with and making meaning. The interviewees talk positively about religion for several reasons: It compensates for their lack of a partner, it allows them to reinterpret their singlehood, or it boosts their self-esteem.

#### Subtheme 1. A Supportive Perspective on Seeing God and the Community

In the participants’ statements, a relationship with God resembles a relationship with another person or a friend, which leads to a reduced sense of loneliness compared with not seeing this type of relationship. For example, Klara, who is a 47-year-old divorcee, notes, “I do talk to God. I ask, I thank, I say good morning,” while 19-year-old single Penelopa who has never been in a relationship says, “When I sometimes feel lonely, I go for a walk and talk to Him.”

The participants also report that religious communities are more likely to be seen as places with various types of support than as places in which to find a partner. Pamela, who is 25 and has never been in a relationship, speaks candidly about this as follows: “For me, a person who is closed off and has relationship problems, the church and my faith have given me such a safe place where I can establish relationships; I will feel safe, and no one will push me away.”

#### Subtheme 2. Questioning the Duty of Being in a Romantic Relationship

This subtheme refers to religious beliefs that allow life in a romantic relationship to be interpreted as something optional and less important than other spheres of life. It also includes religious beliefs, which allow singles to focus on spiritual goals while distancing themselves from problematic singlehood.

The view that being involved in a romantic relationship is not necessary for a Christian is well illustrated by the following statement made by 30-year-old, never-married Kasjan:

I mean, redeemed we already are, but the direction is one—God [salvation]—and we should live according to all His rules and in harmony with others; but apart from the fact that the direction is one, these ways are very many [in terms of life’s paths like singlehood or being in a relationship].

During the interview, Kornel, a 28-year-old who has never been in a relationship, explains that he relates to biblical examples. This allows him to accept his single life. In his argument, he mentions Jesus, who is the central figure of Christianity:

He didn’t have a wife; He just had a circle of friends. So it seems to me that this is an issue where the Lord God leaves the freedom of choice to man; it used to be more social, there was such pressure that someone had to get married, find a wife, and if he didn’t want to, well, he had to become a clergyman. Currently, it’s so much an open question that one can remain neither here nor there and still do some work in the Church, so it’s such an opening for single people.

#### Subtheme 3. The Meaning Provided by Religion in Singlehood

This subtheme centers around the meaning provided by religion in singlehood. It refers to the participants’ religious beliefs that help them understand and accept their situation of living as a single while motivating them to act or not act, as well as redefining their expectations of life.

For example, Ksenia, who is 31 and has never been married, talks about giving meaning to her singlehood as an opportunity given to her by God to correct aspects of her personality that may be a problem in a future relationship:

He [God] does this [keeps us in singlehood] out of love for us because He wants the best for us, and I want to trust in that; I think He just knows that I just need a little more time to work through some of my things so that I can enter into a relationship.

Konrad, who is 32 years old and has never been married, thinks along similar lines; however, he sees his time living as a single as God-given to develop his artistic talent (*Maybe God feels that singlehood will be better for me*). In turn, 35-year-old Kazimiera, who has never been married, clearly describes her sense of invitation from God to help others as follows:

I do not have a partner yet to serve the Lord God more fully with, to go to church with, to read with, and to accompany the kids from the parish somewhere with. I am still significantly involved in parish life, which is probably why the Lord God has not given me this chosen one yet.

This subtheme also shows how important it can be for those interviewed to know their vocation in life, which they understand as God’s will for them manifested in their deep desires (if this search mainly involves suffering, see Subtheme 2 of Theme 1). Finding one’s vocation is usually associated with feelings of peace and fulfillment. This is well and succinctly reflected in the statement of 35-year-old, never-married Grazyna, who states:

I think that there is a principle that God always wants to always want us to be happy. Furthermore, if, for instance, at this point in my life, not having a partner, I feel good, good with myself, well, that is also, in a sense, what God wants.

### Theme 3. Religion as a Criterion for Choosing a Life Partner and the Foundation of an Intimate Relationship

The third theme captures participants’ intention to find a partner who shares their Christian values, along with the belief that God plays an active role in this process. In this context, religion functions not as a coping strategy, but as a guiding framework for life. It shapes their relational orientations, with spiritual identity providing coherence and completeness, thereby reducing perceived pressure to enter a romantic relationship.

Choosing a partner with a particular worldview results from professing religious principles. Nevertheless, it is also dictated by the desire to spend future time together engaged in religious activities or discussions on faith-related topics. The operation of the rule of similarity and the desire to have as many points in common as possible with a partner can be seen in the statement of 28-year-old Karina, who has never been in a relationship: “If I were to be in a relationship with someone, I would want it to be a person with values similar to mine in the aspect of faith.”

The need for a Christian partner is also dictated by the desire to incorporate spirituality into relationship building. Kira, who is 27 and has never been in a relationship, mentioned this, highlighting the importance of religious goals in her life: “I would like my relationship to be simply something that leads to God, however, and not away from Him. When two people share similar values, they can pull each other upward toward the Lord God.”

Among many participants, the search for a partner is linked to a strong belief that God will put the right partner in the participant’s path at the right time for the individual. A related statement by 30-year-old, never-married Klaudiusz is as follows: “But I believe that the Lord God, if He wills it, if it is according to His will, will send me a woman, a wife.”

Searching for a suitable partner can sometimes be time-consuming, during which participants consider lowering their expectations of their partner. Sometimes, however, a need for a partner is so essential that it closes the field to significant doubts. For instance, participant, 37-year-old Krystyna, who has never been married, addresses this issue as follows:

I will not get involved with a guy who is divorced and has three children. That’s the impact of faith. Because I’m single, a virgin, I can get married normally, so I don’t reflect on patchwork families. First of all, there are reasons related to church marriage; secondly, the reason is that I think it’s simply the wrong family model.”

In sum, we generated three themes that built around uniting meaning of the role of religion in the lives of single Christian adults (see Table 2). The first theme reveals the participants’ experience of religion as limiting personal choices and causing frustration in a life lived as a single. It discusses the difficulties resulting from the obligation to follow religious rules in romantic and sexual relationships. Additionally, participants perceive God as someone incomprehensible and uninterested in a person’s well-being but who directs specific requirements for entering a relationship. The second theme unveils religion as a resource to help cope with the stress associated with singlehood and find meaning in a single life. This theme reveals that contact with God and the religious community can compensate for lacking a lifetime partner. The last theme depicts religion as a lifestyle that shapes singles’ approach to romantic relationships; it reveals the participants’ need to find a partner with shared Christian values, and the belief in God’s influence on the success of this search also stems from religion.

## Discussion

The reflexive thematic analysis enabled us to develop three key themes that serve as interpretative narratives (Braun & Clarke, 2023), highlighting the primary roles of religion in the lives of Christian singles. Significantly, during our data analysis phase, we found that our data revealed ways of interpreting singlehood in light of God’s involvement and religion, as well as efforts to achieve cognitive control over one’s singlehood. This recurring theme indicated an underlying cognitive process: the desire to comprehend, articulate, and mentally structure the experience of being single, particularly when it feels involuntary or challenging. This concept later guided us in interpreting our findings through the lens of general attribution theory in psychology (Proudfoot & Shaver, 1975; Spilka & Schmidt, 1983; Spilka et al., 1985).

Attribution theory explores how people interpret the causes of events, especially negative or unclear ones, by ascribing meaning and accountability to themselves, external influences, or divine forces (Spilka & Schmidt, 1983). In our study, participants often viewed their single status through a religious lens, perceiving it as part of God’s plan, a spiritual challenge, or an opportunity for personal development. These interpretations can provide psychological benefits, allowing individuals to reframe situations and restore a sense of coherence and control. Therefore, we recognized that attribution theory is a valuable theoretical framework for explaining our findings. It demonstrated how religious beliefs assist Christian singles in cognitive processing and emotionally managing their singlehood.

Furthermore, some of our participants articulated questions and emotional struggles that can be understood in light of the problem of theodicy. Theodicy refers to attempts to reconcile the existence of evil and suffering with belief in a benevolent and omnipotent God (Stump, 2010). While academic philosophy treats theodicy as a structured doctrinal argument, psychological research has adopted the term to refer more broadly to how individuals make sense of suffering within a religious framework (Wilt et al., 2017). This broader use often focuses on everyday beliefs about God’s justice, purpose, or involvement in life’s difficulties (Hall et al., 2018).

In the current investigation, participants raised theodical questions while making sense of their experiences related to prolonged or unwanted singlehood. Some individuals expressed doubts about God’s justice or intentions, while others reaffirmed their faith in divine timing or considered their current situation spiritually significant. This emphasis on theodicy illustrates Christian singles’ challenges as they strive to reconcile personal suffering with their belief in a benevolent and omnipotent God. These personal narratives serve as lived theodicies—individual attempts to validate divine goodness and coherence amid emotional difficulties. By integrating attribution theory and the concept of theodicy, we could explain our findings showing how religious perspectives influence coping mechanisms, core identity processes, and deep existential reflection among single Christian adults.

### Religion as a Source of Negative Experiences Associated with Singlehood

The first theme reflects the participants’ experience of religion as providing explanations that restrict their freedom of choice, assign negative meaning to their singlehood, and diminish their self-esteem. Single individuals often feel powerless in the situation of remaining single. This is because religious rules impose specific choices on them, such as prohibitions against premarital sex and the inability to enter a sacramental marriage after divorce. Some participants perceive single life negatively, viewing it as a punishment or trial sent by God. Others consider themselves inferior in the eyes of God or the religious community because they place greater value on family and married life.

Such perceptions of religion by the participants in our study may also be enhanced by the specificity of the Christian faith and by the Polish cultural context. For example, religious and spiritual struggles revolving around involuntary singlehood and the unmet desire to have a partner may reflect the experience of the theodicy problem (see Ahmadi et al., 2019). This means that single individuals may wonder why God, who is assumed to take care of his children and despite the assertion of “ask, and you shall receive,” would allow one to remain single despite all efforts to find a partner/spouse. This situation is made possible because Christianity is a theistic religion that recognizes that God can intervene in events in people’s lives if He wants to. In addition, the image of God presented in the Old Testament (one of the two parts of the Bible) is sometimes portrayed as vindictive and cruel (Copan, 2011), which may contribute to instilling such an image of God among faithful singles. In turn, perceived difficulties in expressing one’s sexual expression, in addition to the religious principles mentioned, may be influenced by the image of singles as hedonistically orientated, highly sexually active, and avoiding the lasting relationships present in the Polish context (Czernecka, 2008), which religious singles may wish not to confirm.

The content of the first theme resembles other studies that also revealed the difficulties singles face in practicing their faith. Specifically, Adamczyk and colleagues (2024c) constructed in their interviews of four single Roman Catholic Polish adults two themes (difficulties in adherence to religious rules and difficulties in relationship with God) that reflected the experience of single individuals of an internal struggle between religious values and desires in the domain of romantic relationships and the experience of confusion and dissatisfaction with a relationship with God. Additionally, the maladaptive role of religion in the lives of single individuals has also been observed in studies by Himawan et al., (2018a, 2018b), who demonstrated that religiosity can be associated with negative religious coping, that is, seeing God as a punishing figure or pitying yourself for being abandoned by God.

### Religion as an Aid in Mitigating Negative Experiences Associated with Singlehood

In contrast to the first theme reflecting the negative view of religion in reference to singlehood, the second theme captures the fact that single adults by attributing their singlehood to God’s plan do not perceive it as a random or unfortunate event. Instead, they understand singlehood as an intentional part of God’s design, thereby regaining control over their circumstances. This theme includes three subthemes reflecting three different aspects of relating single adults to religion in the context of their singlehood.

The first subtheme, *A supportive perspective on seeing God and the community*, captures the Christian emphasis on building communities. This theme aligns with previous research suggesting that single individuals often turn to religion to find support and fulfill their attachment and belonging needs (Granqvist & Hagekull, 2000; Himawan et al., 2018a). In Poland, various religious initiatives and lay movements, including prayer groups and Bible study circles, create avenues for singles to forge meaningful connections rooted in shared beliefs. These spiritual communities offer support, care, and a sense of identity, which many participants deem important in their everyday lives.

The second subtheme, *Questioning the duty of being in a romantic relationship*, reflects well the category of religious coping, which is religious concentration, i.e., diverting attention from the problem by focusing on religious issues (Pargament et al., 1998). As mentioned in the Introduction, Christianity makes it possible to look at a possible life in a relationship as something less important than salvation. It guarantees that people will no longer be paired off in eternal life anyway. In turn, redirecting attention to spiritual values (e.g., salvation more important than a relationship) may allow individuals to feel free from the pressure of being in a relationship.

Focusing on higher spiritual values—like salvation or personal holiness—can help regulate negative emotions tied to unwanted singlehood. However, this coping strategy may have mixed outcomes. Studies indicate that religious coping can become maladaptive when rituals or beliefs lack personal integration or commitment (Himawan et al., 2018a). In such instances, religion may act as cognitive avoidance, enabling the suppression of painful feelings and sustaining involuntary singlehood by neglecting emotional work (Dickson et al., 2011). Research recommends a reflective, meaning-oriented approach to using religion, encouraging singles to understand their experiences rather than escape them (Himawan et al., 2018a). Here, religion serves not as denial but as a tool for incorporating singlehood into a broader narrative of purpose and identity. Our findings show that while some participants found relief in theological reframing, others used similar concepts as defenses against distress without deeper engagement with their situation.

The third subtheme, *The meaning provided by religion in singlehood*, illustrates that religion provides positive meanings and opportunities that can help reframe singlehood. It suggests interpreting singlehood as a mission or a chance for spiritual growth, ultimately fostering a heightened sense of self-worth. It highlights the possibility of coping with singlehood by employing benevolent religious reappraisal (Pargament et al., 1998).

This subtheme resembles the theme reported in the study by Adamczyk and colleagues (2024a): Redefining *singlehood through religion as a benevolent and advantageous situation.* The authors described their theme as reflecting single Christian adults’ active attempts to understand single status in positive terms, for instance, by acknowledging single life as God’s plan (Adamczyk et al., 2024c). Therefore, our third subtheme provided evidence that religion might be vital when people search for answers to existential questions or face stressful situations (Krok & Zarzycka, 2021). In turn, such a stressful situation may involve singlehood that inclines individuals to search for meaning and purpose through religion (Adamczyk et al., 2024c).

These positive functions of religion as providing meaning for single individuals’ lives were also observed in qualitative interviews performed by Himawan (2020a, 2020b). Specifically, Himawan (2020a, 2020b) revealed that religion can help single people to interpret singlehood from a religious perspective. It also helps them cope with involuntary singlehood and makes their single life meaningful. This is achieved by perceiving their single status as a temporary period willed by God, as a period that can be used to embrace life outside of marriage, and as a period during which conservative socioreligious norms are negotiated with contemporary values.

### Religion as a Criterion for Choosing a Life Partner and the Foundation of an Intimate Relationship

The last theme encompasses religious explanations that help individuals navigate their romantic history, protecting against casual encounters and guiding the selection of a partner, particularly one who shares their values. They infuse relationships with meaning, emphasizing their spiritual dimension rather than being solely based on emotions. Additionally, religious attributions can help preserve one’s self-esteem by dispelling the notion that individuals are “doomed to be single.” Instead, singlehood is seen as a period of waiting for the right partner—one that God has intended for them. However, singles may employ a range of religious attributions. Some of these attributions enhance control, meaning, and self-esteem. Others, however, may impose limitations or restrictions on their choices and expectations.

Prior research demonstrated that religious similarity ranks approximately 12th in importance among 23 factors associated with mate preferences. People prefer religious similarity regarding presence vs. absence and type of religious affiliation (Mahoney, 2010). Adamczyk and colleagues (2024c) also identified the essence of this theme in qualitative interview-based research focused on four Polish single Roman Catholic adults.

Notably, our participants also had religion in reference to singlehood in the form of collaborative religious coping. In other words, the positive function of religion observed in the current study also involved seeking control through a partnership with God in solving a problem (Pargament et al., 1998), such as the lack of a lifetime partner. This aspect of religion also emerged in the study by Adamczyk and colleagues (2024a) on the theme of *Giving up control of one’s singlehood and Finding a partner/spouse to God.* Entrusting the search for a partner to God involves recognizing that divine assistance does not equate to passivity. Instead, fostering a partnership with God necessitates active engagement from the participants in their quest for companionship. This method of coping also involves “pleading for direct intercession” or seeking control indirectly by pleading to God (praying) (see Ahmadi et al., 2019).

Our findings reveal similarities with studies in other religious contexts, such as predominantly Muslim Indonesia (Himawan et al., 2018a, 2018b; Himawan, 2020a, 2020b). These include religion’s role in fostering belonging and perceived support through community participation, as well as interpreting singlehood as part of God’s plan (Himawan et al., 2018a, 2018b; Himawan, 2020a). Additionally, religious singles in Indonesia struggle with following norms about relationships and express concerns about meeting religious expectations in romance (Himawan, 2020b). These parallels suggest shared functions of religion in single individuals’ lives across different traditions. However, we noted that our participants emphasized marrying a compatible partner, while Indonesian respondents focused more on the necessity of marriage, regardless of compatibility (Himawan et al., 2018a, 2018b; Himawan, 2020a). This difference may need further investigation, especially regarding pro-marriage messages in religious systems (Ibrahim & Hassan, 2009) and their impact on singlehood in various cultural contexts.

## Limitations and Future Research Directions

Despite the major strengths of the current investigation, there were notable limitations. The study involved only representatives of the Christian denomination, which precludes the possibility of applying its findings to other religions and denominations. The study was also restricted to context-specific statements describing the associations between religion and singlehood in the conditions specified for Christian single adults living in Poland. We recognize that Polish Catholicism may have characteristics that are distinct from those of Catholicism in other nations. Based on the narratives provided by the participants, we identify two specific aspects that the Polish religious and cultural context may influence.

The strong public presence of religion may foster social integration and the chance to find like-minded partners in religious circles. The low social acceptance of cohabitation without marriage may increase pressure to formalize romantic relationships, affecting how singles view their status and choices. Conversely, the reduced social acceptance of cohabitation outside of marriage; several participants remarked that social expectations surrounding marriage and the relatively limited acceptance of cohabitation without formal marriage might shape how religious singles perceive their singlehood status and navigate potential romantic relationships. Future studies should include cross-cultural and interreligious comparisons, as experiences of singlehood can differ significantly across various religious and societal contexts (Himawan, 2020b; Tarakeshwar et al., 2003).

Second, the individuals who participated in the current investigation were recruited via posting flyers in Facebook groups for believers of Christian denominations, which might have affected the participants’ sociodemographic, singlehood, and religious characteristics. For instance, social media platforms such as Facebook tend to assemble people with lower levels of religious identification (e.g., Davidson & Farquhar, 2014).

Third, the interviews were conducted via online applications, which might have affected the dynamics of the participant–researcher contact and the breadth and depth of the information provided by the participants compared to the face-to-face interview.

Despite this limitation, our qualitative interview-based research may serve as a compass, i.e., a starting point, for subsequent studies (Noyes et al., 2019), both qualitative and quantitative ones, or mixed-method studies. For instance, future qualitative studies may be designed as longitudinal studies to track single individuals and determine changes in employing religion in the experience of singlehood. In turn, quantitative studies may test in more extensive and more diverse samples the theoretical models depicting the links between singlehood and life outcomes from the perspective of various aspects of religion and mechanisms, such as through the lens of religious attributions (Spilka et al., 1985) or religious struggles (Zarzycka et al., 2017).

## Conclusion

In theoretical terms, the current study makes a valuable contribution to the field by revealing that employing religious attributions allows individuals to experience their single status as meaningful. This is true even if the meaningfulness is achieved by making a negative sense of singlehood. Individuals may feel that God has control over their single status and finding a suitable partner in the future. Additionally, they may have a sense that their romantic desires and expectations are guided by the religious values to which they adhere. Moreover, our findings provide further support for the centrality of the search for meaning in the cognitive processes activated in response to negative situations (Matos et al., 2024; Park, 2010). Such situations include the experience of involuntary singlehood, which may be experienced as difficult and stressful (Jackson, 2018).

In methodological terms, we have several reflections on the interview procedure and the analysis of the collected data. First, the performance of the interviews via online applications, despite our initial concerns, turned out to be an effective and fruitful way of data collection. Further, due to performing the bracketing interview and the pilot study, we were able to elaborate an interview guide and a set of questions capable of evoking participants’ rich narratives. Our interview scenario is in its original version or after introducing some modifications in future research examining the issues of religion in reference to adult singlehood. Finally, our investigation has methodological merits due to applying reflexive thematic analysis, integrating perspectives of the psychology of religion and singlehood studies, and employing a collaborative interpretative approach. This lens enabled us to discern psychological, existential, symbolic, and spiritual elements in the participants’ stories. Fusing these complementary viewpoints under a constructivist epistemology, the study illustrates how qualitative research can link different disciplines, resulting in a nuanced, context-aware understanding of personal and religious experiences. This approach may serve as a springboard for future qualitative and quantitative studies exploring the intricate relationships among belief, identity, and social life.

Finally, in practical terms, the obtained results may have practical implications in educational and clinical work with religious single adults. They highlight the necessity of examining an individual’s religious meaning-making, attachment to God, and coping strategies in therapeutic and support environments. These factors can greatly affect how singlehood is perceived and managed. Incorporating them into psychological or pastoral care might improve overall well-being, especially when distress accompanies singlehood. Additionally, current therapeutic frameworks in clinical—like the idea of ambiguous loss in singlehood (Jackson, 2018)—could be enhanced by integrating religious and spiritual viewpoints, thus aligning them more closely with the real experiences of religious individuals.

## Data Availability

The dataset generated and analyzed in the present study is not publicly available to honor the individual privacy of the participants but is partially available from the corresponding author on reasonable request. The entire interview schedule used in the investigation is available at the Open Science Framework [https://tinyurl.com/4y7ywxer].

## References

[CR100] Adamczyk, K., & Trepanowski, R. (2023). Singlehood in Europe: Rates and factors associated with happiness. V&R unipress | Vandenhoeck & Ruprecht.

[CR1] Adamczyk, K., Łyś, A. E., & Trepanowski, R. (2024a). Can spiritual well-being help people be satisfied with singlehood? Exploring the temporal link between spiritual well-being and satisfaction with singlehood. *Journal of Spirituality in Mental Health,**26*(4), 349–367. 10.1080/19349637.2023.2232775

[CR2] Adamczyk, K., Trepanowski, R., Łyś, A. E., & Rogowski, W. (2024b). I say a little prayer for my singlehood: Exploring the role of prayer for single adults’ mental health. *Journal of Beliefs and Values*. 10.1080/13617672.2024.2359788

[CR3] Adamczyk, K., Trepanowski, R., Mrozowicz-Wrońska, M., & Janowicz, K. (2024c). The role of religion in the mental health of single adults: A mixed-method investigation. *International Journal for the Psychology of Religion,**34*(1), 1–23. 10.1080/10508619.2023.2265275

[CR4] Ahmadi, F., Erbil, P., Ahmadi, N., & Cetrez, Ö. A. (2019). Religion, culture and meaning-making coping: A study among cancer patients in Turkey. *Journal of Religion and Health,**58*(4), 1115–1124. 10.1007/s10943-018-0646-7 29872943 10.1007/s10943-018-0646-7 PMC6606655

[CR5] Bäcker, R., Wincławska, M., Rak, J., Czechowska, L., Gadomska, G., Gajda, J., Gawron-Tabor, K., Kasprowicz, D., Mateja, M., Płotka, B., Szewczak, W., & Wojciechowska, J. (2016). *Metodologia badań politologicznych* [Methodology of political science research]. Polskie Towarzystwo Nauk Politycznych.

[CR6] Braun, V., & Clarke, V. (2006). Using thematic analysis in psychology. *Qualitative Research in Psychology,**3*(2), 77–101. 10.1191/1478088706qp063oa

[CR7] Braun, V., & Clarke, V. (2020a). Can I use TA? Should I use TA? Should Inotuse TA? Comparing reflexive thematic analysis and other pattern-based qualitative analytic approaches. *Counselling and Psychotherapy Research,**21*(1), 37–47. 10.1002/capr.12360

[CR8] Braun, V., & Clarke, V. (2020b). One size fits all? What counts as quality practice in (reflexive) thematic analysis? *Qualitative Research in Psychology,**18*(3), 328–352. 10.1080/14780887.2020.1769238

[CR9] Braun, V., & Clarke, V. (2023). Toward good practice in thematic analysis: Avoiding common problems and be(com)ing a knowing researcher. *International Journal of Transgender Health,**24*(1), 1–6. 10.1080/26895269.2022.2129597 36713144 10.1080/26895269.2022.2129597 PMC9879167

[CR10] Braun, V., & Clarke, V. (2025). Reporting guidelines for qualitative research: A values-based approach. *Qualitative Research in Psychology,**22*(2), 1–40. 10.1080/14780887.2024.2382244

[CR11] Catholic Church. (1997). *Catechism of the Catholic Church* (2nd ed.). Libreria Editrice Vaticana. https://www.vatican.va/archive/ENG0015/_INDEX.HTM

[CR12] Centre for Public Opinion Research (CBOS) (2019) Alternatywne modele życia rodzinnego w ocenie społecznej [Alternative models of family life in social evaluation]. Available at: https://www.cbos.pl/PL/publikacje/raporty.php

[CR13] Centre for Public Opinion Research (CBOS). (2024). Religijność Polaków w ostatnich dziesięcioleciach. Komunikat z badań [Religiousness of Poles in recent decades. A survey message] Available at: https://www.cbos.pl/SPISKOM.POL/2024/K_050_24.PDF

[CR14] Ciesielski, M. (2015). Teizm umiarkowany jako integracja teizmu chrześcijańskiego i ateizmu. Próba porównania trzech stanowisk w kwestii istnienia Boga [Moderate theism as an integration of christian theism and atheism. An attempt and a comparison of three approaches to the existence of God]. *Colloquium Wydziału Nauk Humanistycznych i Społecznych,**3*, 19–38.

[CR15] Clarke, V., & Braun, V. (2016). Thematic analysis. *The Journal of Positive Psychology,**12*(3), 297–298. 10.1080/17439760.2016.1262613

[CR16] Copan, P. (2011). *Is God a moral monster?: Making sense of the old testament God*. Baker Books.

[CR17] Creswell, J. W. (2012). *Educational research: Planning, conducting, and evaluating quantitative and qualitative research* (4th ed.). Pearson.

[CR18] Czernecka, J. (2008). Polski singiel: obraz w mediach a autowizerunek [Polish single: media image vs. self-image]. In: E. Malinowska (Ed.), *Stereotypy a rzeczywistość na przykładzie wybranych kategorii społecznych [Stereotypes and reality on the example of selected social categories]* (pp.110–137). Oficyna Wydawnicza TERCJA.

[CR19] Davidson, T., & Farquhar, L. K. (2014). Correlates of social anxiety, religion, and Facebook. *Journal of Media and Religion,**13*(4), 208–225. 10.1080/15348423.2014.971566

[CR20] DePaulo, B. M., & Morris, W. L. (2005). Singles in society and in science. *Psychological Inquiry,**16*(2–3), 57–83.

[CR21] Dickson, K. S., Ciesla, J. A., & Reilly, L. C. (2011). Rumination, worry, cognitive avoidance, and behavioral avoidance: Examination of temporal effects. *Behavior Therapy,**43*(3), 629–640. 10.1016/j.beth.2011.11.002 22697450 10.1016/j.beth.2011.11.002

[CR22] George, L. S., & Park, C. L. (2016). Meaning in life as comprehension, purpose, and mattering: Toward integration and new research questions. *Review of General Psychology,**20*(3), 205–220. 10.1037/gpr0000077

[CR23] Girme, Y. U., Park, Y., & MacDonald, G. (2023). Coping or thriving? Reviewing intrapersonal, interpersonal, and societal factors associated with well-being in singlehood from a within-group perspective. *Perspectives on Psychological Science,**18*(5), 1097–1120. 10.1177/17456916221136119 36534959 10.1177/17456916221136119 PMC10475216

[CR24] Granqvist, P., & Hagekull, B. (2000). Religiosity, adult attachment, and why “singles” are more religious. *International Journal for the Psychology of Religion,**10*(2), 111–123. 10.1207/S15327582IJPR1002_04

[CR25] Hall, M. E. L., Shannonhouse, L., Aten, J., McMartin, J., & Silverman, E. (2018). Theodicy or not? Spiritual struggles of evangelical cancer survivors. *Journal of Psychology and Theology,**47*(4), 259–277. 10.1177/0091647118807187

[CR26] Hennink, M. M., Kaiser, B. N., & Marconi, V. C. (2017). Code saturation versus meaning saturation: How many interviews are enough? *Qualitative Health Research,**27*(4), 591–608. 10.1177/1049732316665344 27670770 10.1177/1049732316665344 PMC9359070

[CR27] Hill, P. C., & Edwards, E. (2013). Measurement in the psychology of religiousness and spirituality: Existing measures and new frontiers. In K. I. Pargament, J. J. Exline, & J. W. Jones (Eds.), *APA handbook of psychology, religion, and spirituality (Vol 1): Context, theory, and research* (pp. 51–77). American Psychological Association.

[CR28] Himawan, K. K. (2020a). The single’s struggle: Discovering involuntary singleness in Indonesia through gender and religious perspectives. *The Family Journal: Counseling and Therapy for Couples and Families,**28*(4), 379–389. 10.1177/1066480720950419

[CR29] Himawan, K. K. (2020b). Singleness, sex, and spirituality: How religion affects the experience of being single in Indonesia. *Mental Health, Religion & Culture,**23*(2), 204–215. 10.1080/13674676.2020.1767555

[CR30] Himawan, K. K., Bambling, M., & Edirippulige, S. (2018a). What does it mean to be single in Indonesia? Religiosity, social stigma, and marital status among never-married Indonesian adults. *SAGE Open,**8*(3), 2158244018803132. 10.1177/2158244018803132

[CR31] Himawan, K. K., Bambling, M., & Edirippulige, S. (2018b). Singleness, religiosity, and the implications for counselors: The Indonesian case. *Europe’s Journal of Psychology,**14*(2), 485–497. 10.5964/ejop.v14i2.1530 10.5964/ejop.v14i2.1530 PMC6016032 30008958

[CR32] Himawan, K. K., Bambling, M., Underwood, M., & Edirippulige, S. (2024). In the absence of marriage: Social and religious-based relationships as alternatives to marriage for never-married adults in Indonesia. *Journal of Spirituality in Mental Health,**26*(4), 396–420. 10.1080/19349637.2023.2253443

[CR33] Ibrahim, R., & Hassan, Z. (2009). Understanding singlehood from the experiences of never-married Malay Muslim women in Malaysia: Some preliminary findings. *European Journal of Social Sciences,**8*(3), 395–405.

[CR34] Jackson, J. B. (2018). The ambiguous loss of singlehood: Conceptualizing and treating singlehood ambiguous loss among never-married adults. *Contemporary Family Therapy,**40*, 210–222. 10.1007/s10591-018-9455-0

[CR35] Janicka, I. L., & Szymczak, W. (2019). Can close romantic relationships last? The commitment of partners in married and cohabitant couples. *Current Issues in Personality Psychology,**7*(3), 203–211. 10.5114/cipp.2019.86129

[CR36] John Paul II. (1988). *Christifideles Laici.* https://www.vatican.va/content/john-paul-ii/en/apost_exhortations/documents/hf_jp-ii_exh_30121988_christifideles-laici.html

[CR37] Kirkpatrick, L. A. (2005). *Attachment, evolution, and the psychology of religion*. Guilford Press.

[CR38] Krok, D., & Zarzycka, B. (2021). Interpersonal forgiveness and meaning in life in older adults: The mediating and moderating roles of the religious meaning system. *Religions,**12*(1), 37. 10.3390/rel12010037

[CR39] Lianda, T. C. R., & Himawan, K. K. (2022). A source of hope whilst in waiting: The contributions of religiosity to the psychological well-being of involuntarily single women. *ANIMA Indonesian Psychological Journal,**37*(2), 244–267. 10.24123/aipj.v37i2.5029

[CR40] Mahoney, A. M. (2010). Religion in families 1999 to 2009: A relational spirituality framework. *Journal of Marriage and Family,**72*(4), 805–827. 10.1111/j.1741-3737.2010.00732.x 22102761 10.1111/j.1741-3737.2010.00732.x PMC3219420

[CR41] Mąkosa, P., & Adamczyk, T. (2024). Przemiany religijności w społeczeństwie polskim [Transformations of religiosity in Polish society]. *Verbum Vitae,**42*(1), 1–15. 10.31743/vv.17095

[CR42] Malterud, K., Siersma, V. D., & Guassora, A. D. (2016). Sample size in qualitative interview studies: Guided by information power. *Qualitative Health Research,**26*(13), 1753–1760. 10.1177/1049732315617444 26613970 10.1177/1049732315617444

[CR43] Mathras, D., Cohen, A. B., Mandel, N., & Mick, D. G. (2015). The effects of religion on consumer behavior: A conceptual framework and research agenda. *Journal of Consumer Psychology,**26*(2), 298–311. 10.1016/j.jcps.2015.08.001

[CR44] Matos, L., Park, C. L., Indart, M. J., & Leal, I. (2024). “It’s the God factor”: A qualitative study of Syrian Muslims’ postwar religious meaning-making. *Psychology of Religion and Spirituality,**16*(2), 163–172. 10.1037/rel0000505

[CR45] McGrath, C., Palmgren, P. J., & Liljedahl, M. (2018). Twelve tips for conducting qualitative research interviews. *Medical Teacher,**41*(9), 1002–1006. 10.1080/0142159x.2018.1497149 30261797 10.1080/0142159X.2018.1497149

[CR46] Miles, M. B., Huberman, A. M., & Saldaña, J. (2020). *Qualitative data analysis: A methods sourcebook* (4th ed.). Sage.

[CR47] New International Version. (2011). *Holy Bible, New International Version*. Zondervan.

[CR48] Noyes, J., Booth, A., Moore, G., Flemming, K., Tunçalp, Ö., & Shakibazadeh, E. (2019). Synthesising quantitative and qualitative evidence to inform guidelines on complex interventions: clarifying the purposes, designs and outlining some methods. *BMJ Global Health,**4*(1), e000893. 10.1136/bmjgh-2018-000893 30775016 10.1136/bmjgh-2018-000893 PMC6350750

[CR49] Ochnik, D., & Adamczyk, K. (2025). Exploring the factors associated with well-being and ill-being among single adults: A network analysis. *Journal of Social and Personal Relationships.* Manuscript in review.

[CR50] Okruszek, Ł, Piejka, A., & Żurek, K. (2022). Take me to (the empty) church? Social networks, loneliness and religious attendance in young Polish adults during the COVID-19 pandemic. *Journal of Religion and Health,**61*, 722–740. 10.1007/s10943-021-01486-1 35041126 10.1007/s10943-021-01486-1 PMC8764885

[CR51] Oliver, D. G., Serovich, J. M., & Mason, T. L. (2005). Constraints and opportunities with interview transcription: Towards reflection in qualitative research. *Social Forces,**84*(2), 1273–1289. 10.1353/sof.2006.0023 10.1353/sof.2006.0023 PMC1400594 16534533

[CR52] Pargament, K. I. (2010). Religion and coping: The current state of knowledge. In S. Folkman (Ed.), *The oxford handbook of stress, health, and coping* (pp. 269–287). Oxford University Press.

[CR53] Pargament, K. I., Smith, B. W., Koenig, H. G., & Perez, L. (1998). Patterns of positive and negative religious coping with major life stressors. *Journal for the Scientific Study of Religion,**37*(4), 710–724. 10.2307/1388152

[CR54] Park, C. L. (2010). Making sense of the meaning literature: An integrative review of meaning making and its effects on adjustment to stressful life events. *Psychological Bulletin,**136*(2), 257–301. 10.1037/a0018301 20192563 10.1037/a0018301

[CR55] Piwko, A. M. (2013). Chrześcijańskie rozumienie małżeństwa [The marriage in Christianity]. *Warszawskie Studia Pastoralne,**3*(20), 141–169.

[CR56] Proudfoot, W., & Shaver, P. (1975). Attribution theory and the psychology of religion. *Journal for the Scientific Study of Religion,**14*(4), 317. 10.2307/1384404

[CR57] Saroglou, V. (2011). Believing, bonding, behaving, and belonging. *Journal of Cross-Cultural Psychology,**42*(8), 1320–1340. 10.1177/0022022111412267

[CR58] Savin-Baden, M., & Howell-Major, C. (2013). *Qualititative research: The essential guide to theory and practice*. Routledge.

[CR59] Spilka, B., & Schmidt, G. (1983). General attribution theory for the psychology of religion: The influence of event-character on attributions to God. *Journal for the Scientific Study of Religion,**22*(4), 326–339. 10.2307/1385771

[CR60] Spilka, B., Shaver, P., & Kirkpatrick, L. A. (1985). A general attribution theory for the psychology of religion. *Journal for the Scientific Study of Religion,**24*(1), 1–20. 10.2307/1386272

[CR61] Spilka, B., Shaver, P. R., & Kirkpatrick, L. A. (1997). A general attribution theory for the psychology of religion. In B. Spilka & D. N. McIntosh (Eds.), *The psychology of religion: Theoretical approaches* (pp. 1535–2170). Westview Press.

[CR62] Stump, E. (2010). *Wandering in darkness: Narrative and the problem of suffering*. University Press.

[CR63] Tarakeshwar, N., Stanton, J., & Pargament, K. I. (2003). Religion: An overlooked dimension in cross-cultural psychology. *Journal of Cross-Cultural Psychology,**34*(4), 377–394. 10.1177/0022022103034004001

[CR64] Tsang, J.-A., & McCullough, M. E. (2003). Measuring religious constructs: A hierarchical approach to construct organization and scale selection. In S. J. Lopez & C. R. Snyder (Eds.), *Positive psychological assessment: A handbook of models and measures* (pp. 345–360). American Psychological Association.

[CR65] Wilt, J. A., Exline, J. J., Lindberg, M. J., Park, C. L., & Pargament, K. I. (2017). Theological beliefs about suffering and interactions with the divine. *Psychology of Religion and Spirituality,**9*(2), 137–147. 10.1037/rel0000067

[CR66] Zarzycka, B. (2009). Tradition or charisma. Religiosity in Poland. In B. Stiftung (Ed.), *What the world believes: Analysis and commentary on the Religion Monitor 2008* (pp. 201–222). Verlag Bertelsmann Stiftung.

[CR67] Zarzycka, B., Rybarski, R., & Śliwak, J. (2017). The relationship of religious comfort and struggle with anxiety and satisfaction with life in roman catholic Polish men: The moderating effect of sexual orientation. *Journal of Religion and Health,**56*(6), 2162–2179. 10.1007/s10943-017-0388-y 28343284 10.1007/s10943-017-0388-y PMC5653706

[CR68] Żemojtel-Piotrowska, M., Piotrowski, J., & Sawicki, A. (2023). Religiosity, spirituality, national narcissism, and prejudice toward refugees and sexual minorities in Poland. *Psychology of Religion and Spirituality,**15*(4), 533–542. 10.1037/rel0000430

